# The House Fly as a Disease Carrier*Read before the Parker-Palo Pinto County Medical Society, August 10, 1909.

**Published:** 1909-10

**Authors:** Alexander S. Garrett

**Affiliations:** Springtown, Texas


For Texas Medical Journal.

### The House Fly as a Disease Carrier.*

BY ALEXANDER S. GARRETT, M. D., SPRINGTOWN, TEXAS.

The common house fly, known as the species "Musca domestica," according to recent investigations and reports, is considered one of the most frequent and common carriers of infectious diseases. Of so much and so great importance has the house fly been adjudged in carrying disease germs to healthy individuals, thereby infecting them, I have been impressed that a paper on this subject would not be inappropriate.

Whether or not the common house fly that we have in our midst now is the same species or some of the same species that were sent upon the Egyptians we do not know. Referring to the plague of flies, the Bible says in one place, "the land was corrupted by reason of the swarms of flies," and in another place "He sent divers sorts of flies among them, which destroyed them"; while the author of the Book of Ecclesiastes says, "Dead flies cause the ointment of the apothecary to send forth a stinking savor." The Bible is not held to be authority on entomology in reference to the fly, yet it gives some idea of the damaging effect flies had upon the Egyptians during the reign of Pharaoh, King of Egypt.

The breeding place of the house fly, or rather as Professor L. O. Howard, entomologist and chief of the bureau at Washington would term it, "the typhoid fly," is mostly in horse manure. But it also breeds in human excrement and in decaying animal and vegetable matter of various kinds where there is heat and fermentation. According to the most recent investigations a single female fly lays on an average one hundred and twenty eggs during the summer season. These eggs hatch and develop into mature flies in about ten days. At this rapid rate of multiplying it has been estimated that a single fly will have a progeny running up to the sixtillions at the end of a summer season. It is no wonder, then, that flies should be so numerous in summer when the conditions for their increase and development are so favorable.

In 1900, Professor L. O. Howard, of Washington, made an investigation of flies collected in the dining rooms in different parts of the country in reference to species, and found that out of a total of 23,087 flies 22,808, or 98.8 per cent of the whole number, were

*Read before the Parker-Palo Pinto County Medical Society, August 10, 1909.

the house or "typhoid fly." This demonstrates plainly that it is the house fly chiefly that we have to deal with in reference to disease.

In studying the most recent available literature upon the subject of the house fly as a disease carrier, I have found plenty of circumstantial evidence to convince the most skeptical that he is an active agent in the distribution of disease germs, carrying them upon his feet, legs, body, proboscis and in his digestive organs; depositing the same upon food of all kinds, and in milk, water, tea, coffee and the table beverages, and on the persons of those in good health by which means of depositing the deadly disease germs much sickness, suffering, financial loss and death result to the human race. That flies can carry what would appear to be a sufficient number of bacteria to produce infection was demonstrated by investigation made at the Storrs Agricultural Experiment Station, Storrs, Conn., and published in Bulletin No. 51, April, 1908, entitled "Sources of Bacteria in Milk," by W. M. Esten and C. J. Mason. In an examination of 414 flies gathered from different sources of filth, was found that the number of bacteria on each fly run from 550 to 6,600,000, or an average of more than 1,250,000 bacteria on each fly.

I am not prepared to say just how many bacteria it would require to produce infection, but it appears to me that a few full doses from a fly with a million or more bacteria under favorable conditions would be sufficient. Much has been written about the diet and manners of the fly. They appear to be entirely void of manners or politeness, and have a taste for everything clean and unclean, good of bad, cooked or uncooked, sound or rotten, with a special fondness for objects rotten and unclean. They will come from the putrescent and most loathsome objects of filth that ever offended the nose of man, and light upon the savory viands and delicacies of the festive board, sup tea from the same cup that serves the host and hostess, make a door mat of the innocent babe's ruby lips on which to wipe his vile feet, mixes human excrement with the nectar of beauty.

Dr. C. V. Chapin, Health Officer of Providence, R. I., says: "Flies are filthy insects. They drink from the cesspools, dine in the privy vaults, feast on the sputum on the sidewalks, and revel in the garbage pail. They swarm on the baby's diaper, and are greedy in consuming the excreta discharged by old wounds and chronic sores."

As far back as 1849 flies were believed to be connected with carrying disease. Quoting from Bulletin No. 78, U. S. Department of Agriculture, page 27, Professor L. O. Howard says: "One of the earliest accurate scientific studies of the agency of insects in the transfer of human diseases was in regard to the fly as a potential agent in the dissemination of cholera. The belief in this agency long preceded its actual proof. Dr. G. E. Nichols, in the *London Lancet,* Vol. 2, 1873, page 724, is quoted by Nutall as writing as follows regarding the cholera prevailing in Malta in 1849: 'My first impressions of the possibility of the transfer of the disease by flies was derived from the observation of the manner in which these voracious creatures, present in great numbers, and having equal access to the defections and food of patients, gorged themselves indiscriminately, and then disgorged themselves on the food and drinking vessels. In 1850 the *Superb,* in common with the rest of the Mediterranean squadron, was at sea for nearly six months; during the greater part of the time she had cholera on board. On putting to sea the flies were in great force, but after a time the flies disappeared, and the epidemic gradually subsided. On going into Malta harbor, but without communicating with the shore, the flies returned in greater force and the cholera also with increased violence. After more cruising at sea the flies disappeared gradually with the subsidence of the disease.' "

It seems that this important subject suggested as it was many years ago never received much attention until the American-Spanish War, in 1898, when there was a terrible scourge of typhoid fever in the camps of the American soldiers. The great prevalence of typhoid fever in the camps, and especially at Chickamauga, caused the surgeons to make very close observations in reference to the spread of the disease among the soldiers. Quite a number of the army surgeons having observed that flies were numerous in the camps, especially where typhoid fever was most prevalent, and that the disease abated at the coming on of cold weather that checked the multiplication and spread of flies, and that the officers whose tents were screened did not have the disease so much, while the private soldiers who occupied unscreened tents suffered largely from typhoid, rendered it as their opinion, which is found in the government reports on the Origin and Spread of Typhoid Fever in the U. S. Military Camps During the Spanish War of 1898, Vol. 1, that flies were one of the causes, and some believing the chief cause of the spread of typhoid fever among the troops. Since the publication of the report of 1904, other investigations of note have been made in reference to the house fly as a disease carrier in general.

A vast amount of useful information upon the subject has recently appeared, coming from different sources, prominent among which are the reports of Dr. L. O. Howard, Chief of Bureau of Entomology, and a booklet of forty-eight pages recently issued by the Merchants Association of New York, entitled "The House Fly at the Bar; Indictment—Guilty or Not Guilty?"

In the Spanish War Report, on page 660, the statement is made: "Flies swarmed over infected fecal matter in the pits and then visited and fed upon the food prepared for the soldiers at the mess tent. In some instances where lime had been recently sprinkled over the contents of the pits, flies with their feet whitened with lime were observed walking over the food. It is possible for the fly to carry the typhoid bacillus in two ways. In the first place, the fecal matter containing the typhoid germ may adhere to the fly and be mechanically transported; in the second place it is possible for the typhoid bacillus to be carried in the digestive organs of the fly and deposited with its excrement. Typhoid fever bacilli have been identified by culture tests from flies caught in two undrained privies, from the fences of two yards, the walls of two houses, and the room of a typhoid patient." (Spanish War Reports on Typhoid Fever, page 600.)

Major Dellinger, Surgeon-General Third North Carolina Volunteers Infantry, says that he was quite thoroughly convinced that the chief agent in the spread of typhoid fever was the fly. The water supply at Knoxville, he says, was good. Major Clark, Major and Surgeon Twelfth Minnesota, says: "The chief cause of typhoid fever at Chickamauga Park was, without question of doubt, the flies and dust." Walter S. Stewart, Major and Surgeon Ninth Pennsylvania Volunteer Infantry, Spanish-American War, says: "One of the strong arguments in favor of flies as a cause of contagion was that very rarely did one find a case of fever among the staff officers which could be explained only by the fact that they all had their mess tents carefully screened." Reporting on the same subject, Lieutenant Mowery, Assistant Surgeon First Illinois Volunteer Cavalry, makes this statement: "From personal observation I believe that the fever epidemic was due much more to flies than to water." Lieutenant Cox, of the Sixth U. S. Volunteers Infantry, also expresses his opinion to the effect "that the most patent cause of typhoid fever at Chickamauga was the great number of flies which alternately visited privy vaults and mess tents. Time and again I saw flies with lime on them fresh from the vaults."

Many more reports and statements from competent and unquestioned authority could be added verifying the conclusions of the army surgeons in the Spanish-American War that the fly was one of the principal agencies in the spread of typhoid fever in the army.

It will be noted, however, that not all of the surgeons reported the fly as a carrier of disease. Some reported the dust, hands, clothing and the fly together as carriers of the typhoid germs.

The foregoing quotations are taken from the Report on the Origin and Spread of Typhoid Fever, U. S. Military Camps During the Spanish War of 1898.

"The House Fly at the Bar," issued by the Merchants Association of New York, gives many instances in which it is believed, if not positively known, that the house fly was responsible for epidemics of typhoid fever. The reports and information in the little booklet was obtained from health officers, physicians and other authorities throughout the United States and Canada.

Dr. W. C. Hassler, Chief Sanitary Inspector, San Francisco, reports that experiments conclusively prove that the house fly was responsible for practically one hundred and fifty cases of typhoid fever subsequent to the earthquake.

H. O. Braggerman, M. D., Secretary of the Board of Health, Fort Wayne, Indiana, states in his official report as follows: "We have been able to trace one small epidemic of typhoid fever to a city dairy where, in our opinion, the milk was infected by flies which had received the infected organism from an overflowing vault."

Dr. S. J. Crumbine, Secretary Kansas State Health Board, gives in his evidence against the fly, under date November 9, 1908, the following testimony: "The Health Department of the State of Kansas a year previous to the above date undertook the investigation of the cause of typhoid fever that prevailed at an alarming extent in a number of the smaller towns in the western part of the State, and the conclusion arrived at was that the house fly or 'typhoid fly' spread the disease."

Dr. Henry D. Holton, Secretary State Board of Health, Vermont, reports several cases of typhoid infection through the agency of flies; while Dr. J. Glassick, Secretary State Board of Health, South Dakota, reports an epidemic of typhoid fever in a town of fifteen hundred inhabitants, caused by flies, which abated as soon as frost came and killed the flies.

Many other quotations from late reports of unquestionable authority on the house fly as an active agent in causing epidemics of typhoid fever could be added, but I think the foregoing is all sufficient to establish the truth of the theory based as it is on actual conditions.

In view of the fact that there is so much sickness, and that so many deaths occur from typhoid fever, the subject of its cause and prevention becomes one of such momentous consequences, it should engage our most serious thought. I desire to call your attention to the fact that it is being observed that not only does typhoid largely come and go with the advent and departure of flies, but the fly is also charged with the spread of diarrheal diseases not of typhoid type, which come and go alike with the arrival and departure of the flies.

It was estimated that there were in the City of New York, in 1907, 4496 deaths from diarrheal diseases, excluding typhoid fever, and all due to flies, and in 1908 there were 4013 deaths from diarrheal diseases due to the same cause.

Supposing the estimation of deaths in New York City due to the house fly to be correct, and giving that city a population of 3,000,000, and the United States a population of 84,000,000, taking New York City as the standard, though the fly season in the South is much longer than in the North, there occurred 125,798 deaths in the United States in 1907 from diarrheal diseases alone, not including typhoid fever, caused by the house fly. Amazing! Verily we must have the Egyptian plague upon us now.

The death rate in New York City last year from causes stated was much lower because war had been made on the fly.

No wonder that it has been said by high authority: "Regarded in the light of recent knowledge acquired through scientific investigation, the fly is more dangerous than the tiger or the deadly cobra; worse than that, he is, at least, in our climate much more to be dreaded than the mosquito, and may easily be classed the world over as the most dangerous animal on earth."

Not only is it claimed, and undoubtedly proven, that the house fly carries typhoid fever and diarrheal diseases, including dysentery, but tuberculosis, pneumonia, whooping cough and purulent ophthalmia are likewise said to be carried by the same agent.

Dr. Fred J. Mayer, State Lecturer on Hygiene, Mississippi, says: "The ubiquitous unspeakably filthy house fly, feeding on the secretions of the infected, may convey the measles to others and establish a new foci of infection."

So far we have not referred to our own observations in reference to typhoid fever and other diseases supposed to be conveyed by the fly. We have taken the testimony of those high in professional standing and those who have made scientific investigation.

In our professional experiences, no doubt the majority of us can call to mind instances in which several members of the same family have had typhoid fever during the same season and close together. Some may have experienced or witnessed epidemics more or less extensive, all of which, except possibly the first case, according to recent investigation and teachings, may have been caused by the house fly.

Fellow physicians, in view of the teachings and facts made known to us about the dangerous infecting agency of the house fly, what is our duty? The Apostle James says: "To him that knoweth to do good and doeth it not, to him it is a sin." It may be your son or daughter, or it may be my son or daughter, who is cut down in early manhood or womanhood by that fearful scourge known as typhoid fever. Or it may be your precious child or my grandchild or your grandchild that is hurried off prematurely to another world by the filthy work of the abominable house fly.

This is appealing to our selfish nature. But true humanitarianism extends to and includes all mankind. The true spirit of medicine at the present day is principally preventive. It is much greater and more noble and humane in the physician to prevent, if possible, a case of sickness than to cure it after it occurs.

Let us take up the battle cry of cleanliness about the premises, screening and disinfecting the privy vaults, and contents of horse stables or horse manure, thorough screening of all cook and dining rooms, and protecting the rooms of the sick from the invasion of the fly; waging a relentless war upon them under the black flag and banner of "no quarters shown" until their malignant power for evil is reduced to its lowest minimum.

May the age of health soon come, and the time "when the earth shall be as full of the knowledge of the Lord (and His beneficent laws) as the waters that cover the sea." And when there will not be any more necessity of the surgeon removing, or temptation to remove, the vermiform appendix; when bitter or nauseating medicines will be no longer needed; when the joys and sorrows, pains and pleasures connected with the practice of medicine, together with the profession of medicine, will have passed away, and the hygienist will have taken the place of the physician to lead the people on to a more perfect and more glorious attainment in physical and spiritual existence.

